# The Life Aquatic at the Microscale

**DOI:** 10.1128/mSystems.00150-17

**Published:** 2018-03-27

**Authors:** Jean-Baptiste Raina

**Affiliations:** a Climate Change Cluster, University of Technology Sydney, Ultimo, NSW, Australia

**Keywords:** early-career researcher, marine microbiology, symbiosis

## Abstract

There are more than one million microbial cells in every drop of seawater, and their collective metabolisms not only recycle nutrients that can then be used by larger organisms but also catalyze key chemical transformations that maintain Earth’s habitability. Understanding how these microbes interact with each other and with multicellular hosts is critical to reliably quantify any functional aspect of their metabolisms and to predict their outcomes on larger scales. Following a large body of literature pioneered by Farooq Azam and colleagues more than 30 years ago, I emphasize the importance of studying microbial interactions at the appropriate scale if we want to fully decipher the roles that they play in oceanic ecosystems.

## Abstract (precis)

There are more than one million microbial cells in every drop of seawater, and their collective metabolisms not only recycle nutrients that can then be used by larger organisms but also catalyze key chemical transformations that maintain Earth’s habitability. Understanding how these microbes interact with each other and with multicellular hosts is critical to reliably quantify any functional aspect of their metabolisms and to predicting their outcomes on larger scales.

## PERSPECTIVE

Microbes have inhabited our planet for at least 4 billion years, and symbioses have been one of the main drivers of evolution. The first eukaryotic cells were derived from an endosymbiosis, and multicellular organisms have ever since maintained persistent and sometimes obligatory relationships with microbes that not only influence their health but also play key ecological roles (1). This is where my research interests lie: deciphering the functional roles of marine microbes, especially in the context of symbiotic interactions. In the marine environment, there is no better place to study symbioses than coral reefs. These ecosystems are sustained by a myriad of mutualistic interactions, whereby dense populations of bacteria, archaea, and unicellular algae inhabit coral tissue and collectively contribute to coral health and reef accretion by powering the metabolically expensive process of coral calcification (2). It has been hypothesized that these numerous mutualistic symbioses arose because coral reefs occur in nutrient “deserts” (3), where very tight recycling of energy and nutrients between primary producers and consumers is necessary to avoid dilution of resources in the surrounding seawater.

Bacteria have long been known to be abundant and metabolically active in coral reefs, but their wide taxonomic diversity was revealed only in the early 2000s, when the first culture-independent studies were carried out on healthy corals (4). It is now known that there are typically 10 to 100 times more bacteria on the surface of corals than in the water surrounding the reefs, and their diversity exceeds thousands of unique “species” (2). However, obtaining a clear understanding of the functional roles that bacteria play in corals is still an ongoing challenge. During my earlier studies, I focused on the role of coral-associated bacteria in the marine sulfur cycle and more specifically, their use of a sulfur compound called dimethylsulfoniopropionate (DMSP). This compound is present in large concentrations in reef-building corals harboring algal symbionts and at the time was thought to be solely produced by photosynthetic organisms. Functionally speaking, DMSP is the chemical equivalent of a Swiss army knife—it is a key nutrient for marine microbes, a signaling molecule attracting organisms ranging from bacteria to fish, an antioxidant, an osmolyte, and even a precursor of a gas involved in cloud formation (5). I investigated the role that DMSP plays as a nutrient for coral-associated bacteria (6) and spent several years developing a method to preserve this water-soluble compound in order to visualize and quantify its exchange between microalgal producers and bacterial consumers (7). In contrast to our initial assumptions, we discovered that DMSP was produced not only by the microalgae inhabiting coral tissue but also by the coral animal itself—this was the first evidence for DMSP production by a nonphotosynthetic organism (8). More recently, the DMSP story has become even more interesting: heterotrophic bacteria have also been identified as DMSP producers (9), shifting a 60-year-old paradigm. Corals are therefore a unique system in which bacteria, unicellular algae, and the animal host might each contribute to the large DMSP concentrations measured in this ecosystem. How this DMSP production is partitioned and the exact function(s) this compound plays in this complex symbiosis are yet to be elucidated.

**FIG 1:**
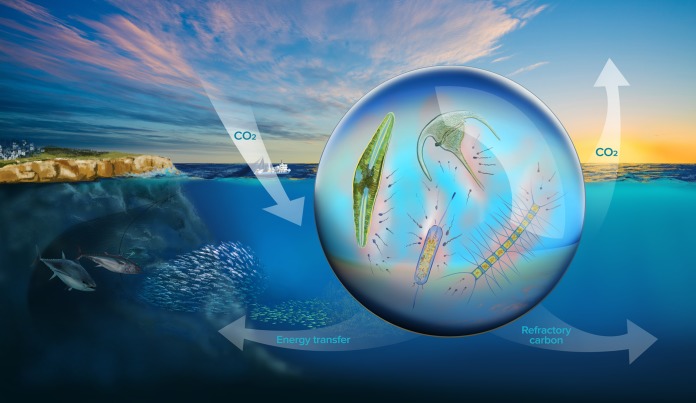
Microscale heterogeneity in the ocean and its potential impacts on the marine carbon cycle. Phytoplankton-bacterium interactions for example (depicted in the central droplet of seawater) might directly influence biogeochemical cycles, energy transfer to higher trophic levels, sequestration of refractory carbon, and release of climate-active gases.

Most marine symbioses have been studied in organisms attached to the seafloor like corals, but the scarcity of resources and nutrients in the pelagic water column is also an ideal setting for microbial symbioses to flourish, and this environment has become the focus of my more recent work. Counterintuitively, at the scale of marine microbes, seawater is not homogenous but instead characterized by hot spots of nutrients on and around microscopic organic particles (or marine snow) and in association with phytoplankton and zooplankton (10). The distance between a bacterial cell in the water column and one of these hot spots can be hundreds of body lengths, which means that to colonize and exploit one of these microscale features, a motile bacterium must extensively explore its local environment. Understanding how cells navigate between these microenvironments and interact with each other is very important, because microbial activity and transformation rates in these nutrient hot spots have been predicted to considerably exceed background levels and may have ecosystem-wide impacts (Fig. 1) (11). However, most of the sampling tools used by microbiologists to sample the marine environment typically collect liters of seawater, averaging out these processes and sometimes even rendering them undetectable. More than 2 decades of work have shown the importance of capturing the microscale dynamics of marine microbes (12), yet so far most studies have been limited to theoretical or laboratory systems. If we are to truly understand the role and impact that microbial interactions have in oceanic ecosystems, we first need to sample them at relevant scales to understand how their spatial structures might influence their metabolism and ecology. As part of a team that includes engineers, physicists, molecular biologists, and microbial ecologists, we are developing new sampling devices to capture and quantify microbial interactions at appropriate scales. Our first platform, called the *in situ* chemotaxis assay (ISCA), mimics diffusive point sources of chemicals and permits selective capture of live microbes from the environment based on their behavior and chemical preferences (13).

**FIG 2:**
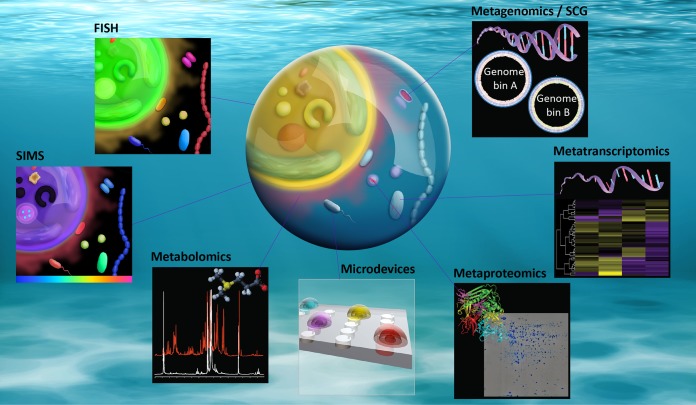
Some of the techniques currently available to untangle marine microbial interactions (such as phytoplankton-bacterium interactions depicted in the central droplet of seawater). The array of tools now available to study microbial systems is impressive, numerous meta-omics can be used to recover the genomes, transcripts, proteins, and metabolites present in a given sample. New microdevices such as the *in situ* chemotaxis assay (depicted here) can sample the natural environment at relevant scales. In addition, *in situ* hybridization techniques (ISH) can decipher the spatial structure of a microenvironment, and coupled with high-resolution imaging, such as secondary ion mass spectrometry, link microbial phylogenetic identity with their metabolic capabilities. Abbreviations: SCG, single-cell genomics; FISH, fluorescent *in situ* hybridization; SIMS, secondary ion mass spectrometry.

The ISCA is composed of an array of wells that can each be filled with a different compound. Once loaded with chemicals, the ISCA is immersed in seawater, and these compounds diffuse out of the wells into the surrounding environment, creating concentration gradients extending up to 2 mm above each well. Chemotactic bacteria within the water column can respond to these cues by swimming into specific wells, with the strength of chemotaxis quantified by flow cytometry. In addition, the identity and metabolic capabilities of the responding microbes can be determined using DNA sequencing approaches. By probing for microbial behavioral traits directly in the environment, the ISCA opens up a new realm of possibilities: for example, it will enable identification of chemicals mediating the acquisition of symbionts and allow for the enrichment and isolation of bacteria capable of metabolizing specific pollutants and the interrogation of bacterial foraging behavior in marine, freshwater, and soil ecosystems. We strived to produce a device that is operationally simple and have recently released a step-by-step protocol of its utilization (https://www.protocols.io/view/fabrication-and-deployment-of-the-in-situ-chemotax-kztcx6n) in order to facilitate its adoption by the scientific community. We hope that the ISCA will quickly become part of the toolkit commonly used to study microbial systems (Fig. 2).

Looking ahead, I aim to use my previous experience with both benthic and pelagic symbioses and my method development skills to study marine symbiotic interactions at ecologically relevant scales in the natural environment. This will enable the integration of spatial complexity into our understanding of microbial ecology, which will be critical to robustly predict how microbial interactions scale up to affect ocean-scale processes. Several technological advances will undoubtedly facilitate my endeavors, including (i) reductions in the amount of starting materials needed for meta-omic analyses. We are currently pushing the limits of low-input metagenomics and metatranscriptomics, with new protocols developed whereby only few hundred cells are needed to obtain reliable metagenomes (14). More than just conveniently enabling scientists to collect smaller-volume samples, this will progressively move our focus from the macroscale to the microscale and generate more robust inferences that consider the effect of microscale heterogeneity rather than averaging it out. (ii) In addition, three-dimensional (3D) printing is coming of age and now allows ideas to be quickly translated into concrete and testable microdevices. We used this technology to manufacture the ISCA, and it shows much potential in the field of microfluidics. The resolution of the best 3D printers is already around 10 µm and is predicted to improve further in the near future. (iii) Finally, recent advances in underwater *in situ* microscopy (15) hold great promises to make the microbial interactions that shape the health of our ocean “visible,” not only to the scientific community but also to the general public.

It is a very exciting time to be a microbial ecologist, as new techniques and methodological advances are rapidly emerging and will progressively allow us to answer previously intractable questions in this field. The power of direct visualization in particular should not be underestimated: many scientific vocations, including mine, started by the astonishment of discovering the unseen through television documentaries. For most people, the word “bacteria” is still a synonym of “disease,” and imaging the central roles microbes play in underpinning the health of their hosts is undoubtedly a key step to change this perception. There is still much to explore and discover by looking at the marine environment through a “microbe’s eye view.”
